# Analysis of postures for handwriting on touch screens without using tools

**DOI:** 10.1038/s41598-021-04367-5

**Published:** 2022-01-07

**Authors:** Sara Marullo, Maria Pozzi, Monica Malvezzi, Domenico Prattichizzo

**Affiliations:** Department of Engineering and Mathematics, University of Siena, Siena, 53100 Italy

**Keywords:** Engineering, Mechanical engineering

## Abstract

The act of handwriting affected the evolutionary development of humans and still impacts the motor cognition of individuals. However, the ubiquitous use of digital technologies has drastically decreased the number of times we really need to pick a pen up and write on paper. Nonetheless, the positive cognitive impact of handwriting is widely recognized, and a possible way to merge the benefits of handwriting and digital writing is to use suitable tools to write over touchscreens or graphics tablets. In this manuscript, we focus on the possibility of using the hand itself as a writing tool. A novel hand posture named FingerPen is introduced, and can be seen as a grasp performed by the hand on the index finger. A comparison with the most common posture that people tend to assume (i.e. index finger-only exploitation) is carried out by means of a biomechanical model. A conducted user study shows that the FingerPen is appreciated by users and leads to accurate writing traits.

## Introduction

Handwriting requires the sense of self and of what is other than the self, proprioception and exteroception, implies a strong hand-eye coordination, and relies on a fine control of the forces exchanged with the writing tool. However, nowadays the possibility of fast typing on keyboards and touchscreens is making this activity more and more rare, leading to a de-materialization of the writing process. What is clearly missing is the spatial perception of the physical medium, the perception of the tool and also the fine tool management. However, as the embodied cognition model enlightens, handwriting is one of the skills that mainly fostered the human cognitive development: Specific brain synaptic connections and areas specialization were set in a complex interrelation, leading to the simultaneous development of physical and mental capabilities, as remarked by several neuroscientists and educationalists^1–3^. This strong connection between mind and body is becoming thinner and thinner, as the growing concerns about dysgraphia highlight^4^. However, the advantages brought in everyday life by mobile devices cannot be ignored and children are nowadays exposed very early to the use of technology^5,6^.

**Figure 1:**
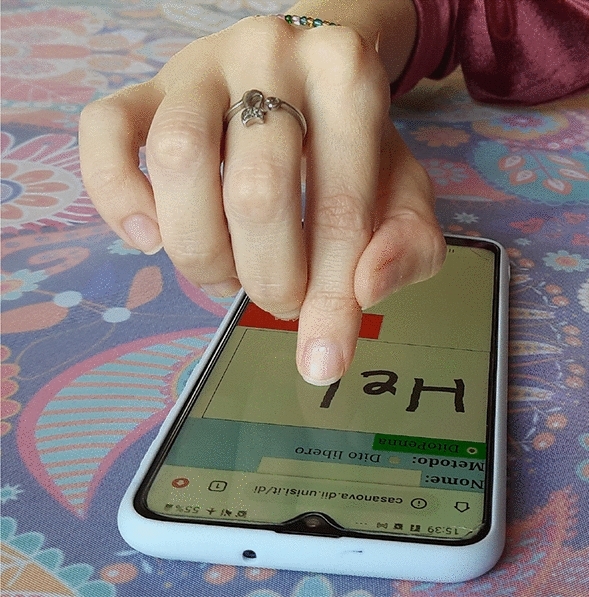
The FingerPen, a hand posture suitable for writing on touchscreens.

According to McLuhan, any technology leads simultaneously to an amputation and a sensorial extension^7^. De Kerckhove investigated also the effects on the human mind, and defined a psychotechnology as “any technology that emulates, extends, or amplifies sensory-motor, psychological or cognitive functions of the mind”^8^. Inspired by these concepts, we propose the FingerPen: a posture of the hand thought to exploit smartphones and tablets to keep people used to practise the “language by hand”^9^. In the FingerPen posture, the index finger is constrained by the hand in such a way that it becomes a writing tool (Fig. 1) characterized by the fact that “tool and gesture merge into a single organ”, similarly to what the anthropologist Leroi-Gourhan observed in animals^10^, leading to an intrinsic embodiment of the tool^11,12^.

Notwithstanding that the fine sensorimotor skills required by the classic handwriting cannot be acquired in other ways except by means of pen and paper, the FingerPen can be exploited in parallel to make children familiarize with the writing task and develop reading-writing prerequisite skills. Berninger said that “We use our hands to access our thoughts”, and showed that children tend to write more quickly, with more words and more ideas while writing by hand than by keyboard^13^. Since tablets are quite spread in all the age groups, some applications of the FingerPen can be proposed for elders, to keep practicing hand-eye coordination and contrast visual-spatial deficit and memory loss, which are amplified by the lack of exercise^14^. Exercises similar to those proposed in the SAGE test^15^, the Clock Drawing test^16^, or the Benton Visual Retention Test^17^ can be performed. Recent studies show that memory benefits from the act of drawing, both in younger and in older adults^18,19^.

### Related works

The handwriting task involves advanced cognitive processes, and is a paradigmatic action that clearly shows human dexterous manipulation capabilities. It would not be possible without the complex structure of the human hand and its underlying sensorimotor control paradigms^20–23^.

The study of the human hand biomechanics^20^ has led to the definition of different kinematic models of the human hand, typically characterized by realistic measures of the phalanges and of their relative positioning^24–26^. Usually, the hand is represented as a set of open kinematic chains made of rigid links connected by revolute joints. Each joint represents a degree of freedom (DoF) of the structure. A common choice is to model index, middle, ring and little fingers with four DoFs (three for flexion/extension, and one at the basis for adduction/abduction)^25,27^, whereas the most used representations of the thumb either include four^25,27^ or five DoFs^28^. When it comes to modeling the handwriting posture, not only the kinematic model of the human hand has to be considered, but also the fact that the writing task starts with a prehensile action^29^, i.e., the achievement of a stable grasp over the writing tool (e.g., a pen). The adopted grasp configuration can affect the handwriting ability, and four main grip styles have been identified based on how fingers are placed around the writing tool^30,31^.

With the advent of touchscreens, researchers have also focused on studying the *digital* handwriting, i.e., the action of writing over a touchscreen either using an ad-hoc tool or just the index finger. With respect to writing on paper, the surface of touchscreens has a lower friction and this might influence the graphomotor execution^32^ and the contribution of different types of sensory feedback (proprioceptive, visual)^33^ during handwriting. Several works investigate digital handwriting in children and older adults by comparing it to other writing methods^34,35^, or focus on similar tasks, like tactile exploration through sliding^36^, but only a few propose a model of the digital handwriting task^37^. In^37^, authors found that using a writing tool allows a more accurate control of the writing action, showing also that the free finger motion is more suitable for tasks requiring a large workspace and small completion time. In this paper, we introduce the FingerPen posture.

## Hand model

To analyze the FingerPen posture from a kinematic point of view, we considered a mechanical model of the human hand in which each finger is an open kinematic chain composed of rigid links connected through revolute joints. In this way, a characterization of the hand operational space can be provided, and the hand in FingerPen posture can be described as a manipulator grasping the distal phalanx of the index finger. The key elements that are needed to model a handwriting posture are: *i)* the links and joints composing the hand, and *ii)* the contact points of the hand with itself and with the environment.

**Figure 2:**
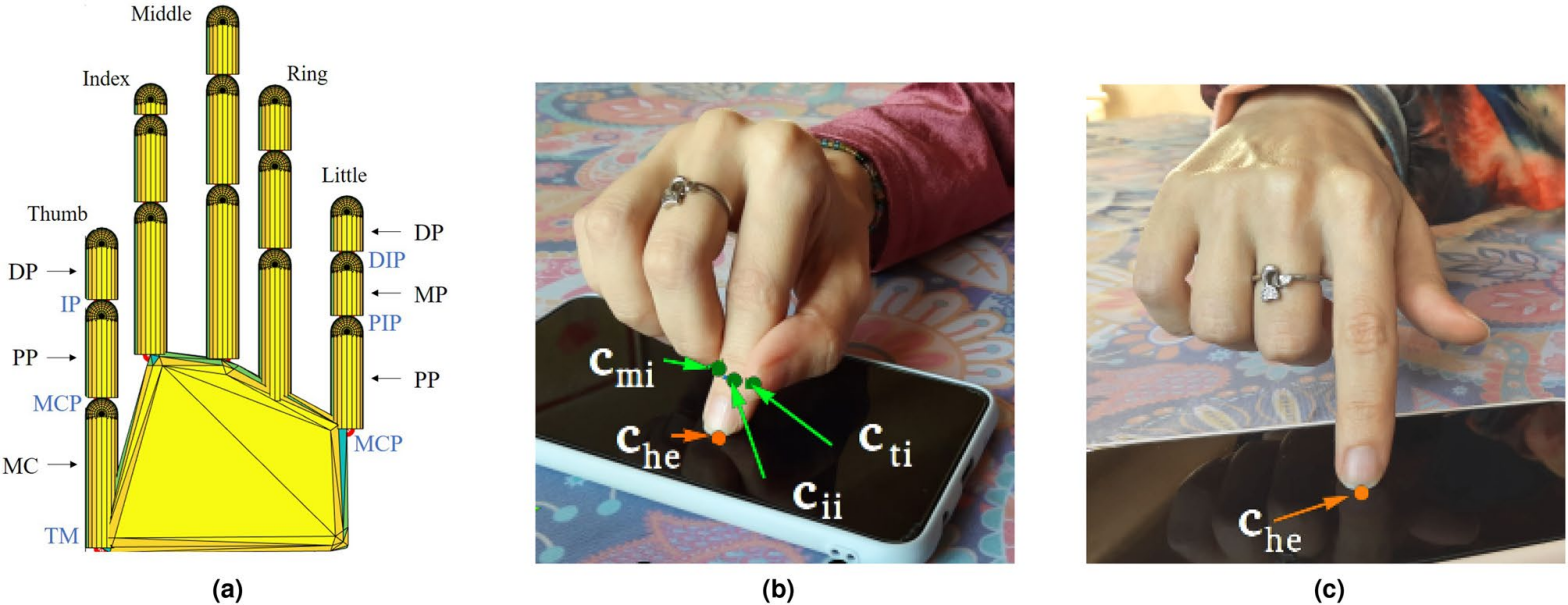
(**a**) Model of the human hand adopted in this work, (**b**) FingerPen contact points. Green arrows indicate the contact points of the hand with itself (i.e., $\mathbf {c}_{hh} = [\mathbf {c}_{ti}, \mathbf {c}_{ii}, \mathbf {c}_{mi}]$), while the orange arrow indicates the contact point between the hand and the environment (i.e., $\mathbf {c}_{he}$). (**c**) In the FreeFinger posture only the contact point with the environment must be considered.

We adopted a 20 DoFs model of the human hand (Fig. 2a) like the one used in^27,37,38^. Index, middle, ring and little fingers are composed of three links, corresponding to proximal (PP), medium (MP) and distal phalanxes (DP), and three joints, corresponding to metacarpophalangeal (MCP), proximal interphalangeal (PIP), and distal interphalangeal (DIP) joints. The thumb has the metacarpal bone (MC), which is connected to the palm through the trapeziometacarpal (TM) joint and to the proximal phalanx (PP) through the metacarpophalangeal (MCP) joint. The proximal and distal phalanges of the thumb are connected through the interphalangeal (IP) joint. The MCP joints of the fingers and the TM joint of the thumb have two degrees of freedom (adduction/abduction and flexion/extension), whereas the other joints only allow flexion/extension movements.

Concerning the contact points, we can indicate with the vector $\mathbf {c}_{hh}$ the position of the contacts between the fingers themselves, and with $\mathbf {c}_{he}$ the position of the contacts between the hand and the environment. In the FingerPen posture (Fig. 2b), the hand has three contact points with itself, whose position is described by the vector $\mathbf {c}_{hh} = [\mathbf {c}_{ti}, \mathbf {c}_{ii}, \mathbf {c}_{mi}]$, where $\mathbf {c}_{ti}$ is the contact point between thumb and index fingers, $\mathbf {c}_{mi}$ is the contact point between index and middle fingers, and $\mathbf {c}_{ii}$ is the contact point between the last phalanx of the index finger and the remaining part of that finger. To properly model the fact that the index tip and the remaining part of that finger are just consecutive parts of the same finger, the last phalanx of the index finger can be constrained (through the adoption of a suitable contact model, see “The FreeFinger posture”) to be completely attached to the index finger itself. Then, the FingerPen establishes a contact point with the environment located on the tip of the index finger ($\mathbf {c}_{he}$).

To better assess the kinematic properties of the FingerPen, in the following we will compare the FingerPen with the FreeFinger posture, i.e., the posture that we typically use to interact with touchscreens, in which the index finger is considered “free”, since it is not constrained by the other fingers ($\mathbf {c}_{hh}=\mathbf {0}$). In this case, the only vector to consider is $\mathbf {c}_{he}$, as the FreeFinger is contacting just the environment (see Fig. 2c). In “The FingerPen posture” and “The FreeFinger posture”, we will provide details on the hand posture for FingerPen and FreeFinger, respectively. By following the analogy with a manipulator, in both cases the index fingertip will be treated as the manipulator end-effector.

In this work, the mathematical vectors representing position and velocity of the contact points of the hand with itself will be denoted with $\mathbf {c}_{hh}$ and $\dot{\mathbf {c}}_{hh}$, respectively, while $\mathbf {c}_{he}$ and $\dot{\mathbf {c}}_{he}$ will denote position and velocity of the contact point between fingertip of the index and the environment. The vectors including the manipulator joint angles and angular velocities will be indicated by $\mathbf {q}$ and $\dot{\mathbf {q}}$, respectively. In the next subsections, we will provide the mathematical relations mapping the hand joint velocities onto the velocity of the index fingertip that interacts with the environment.

### The FingerPen posture

In the FingerPen (FP) posture, the position of the index fingertip is generated by the superposition of the effects caused by the kinematic chains of thumb, index, and middle fingers, while ring and little fingers do not play a role and can be neglected. Since each acting finger is a 4-DoF manipulator, $\mathbf {q}, \dot{\mathbf {q}}\in {\mathbb {R}}^{12}$. By interpreting the FingerPen posture in the light of the grasping theory^39^, we can say that the final part of the index finger is grasped by a manipulator composed by *i)* the kinematic chain of the thumb, *ii)* the kinematic chain of the middle and *iii)* the kinematic chain of the index finger without its final part. As previously mentioned, it is like as if the final part of the index finger has been cut (becoming a separate object) and then grasped by the fingers in specific locations (see Fig. 2b). Moreover, since the fingertip is assumed to remain in contact with the environment and the arrangement of the fingers is such that all the fingers are very close to each other, the angular displacement and velocity potentially provided by the interaction with the thumb and middle fingers can be neglected. Hence, the contact points between these fingers and the index fingertip is represented according to the Hard Finger (HF) contact model^39^. To properly render the fact that the index fingertip is actually attached to the rest of the index finger, the (virtual) contact point of the index with itself is represented according to the Complete Constraint (CC) model^39^.

In the theory of grasping, the contact between the hand and the object occurs when specific locations on the hand and on the object coincide. More specifically, it occurs when the velocity of these locations on the hand and on the object are the same (according to the given contact model). This argument is the basis of the theory on quasi-static grasps^39^, that we applied in the following. The velocity of the contact points on the hand is related to the hand joints by $\dot{\mathbf {c}}_{hh} = \mathbf {J}\dot{\mathbf {q}}$, where $\mathbf {J}\in {\mathbb {R}}^{12 \times 12}$ is the Jacobian matrix of the thumb, index and middle fingers. During the grasp, the velocity of the contact points on the hand is related to the velocity of the grasped object (i.e. the index fingertip) by $\dot{\mathbf {c}}_{hh} = \mathbf {G}^T \dot{\mathbf {c}}_{he}$, where $\mathbf {G}\in {\mathbb {R}}^{3 \times 12}$ is the Grasp matrix. Hence, $\mathbf {J}\dot{\mathbf {q}} = \mathbf {G}^T \dot{\mathbf {c}}_{he}$ that leads to $\dot{\mathbf {c}}_{he} = (\mathbf {G}^T)^{\#}\mathbf {J}\dot{\mathbf {q}}$ where $(\mathbf {G}^T)^\#$ denotes the pseudoinverse of $\mathbf {G}^T$ and it can be proved that $(\mathbf {G}^T)^\#=(\mathbf {G}\mathbf {G}^T)^{-1}\mathbf {G}$. For notation simplicity, the last relation can be rewritten as

1 $$\begin{aligned} \dot{\mathbf {c}}_{he} = \mathbf {J}_{eq} \dot{\mathbf {q}}, \quad \mathbf {J}_{eq} = (\mathbf {G}\mathbf {G}^T)^{-1}\mathbf {G}\mathbf {J}. \end{aligned}$$

In our simulation, the HF contact point on the thumb is located at 3/4 of the length of the distal phalanx, while the HF contact point on the middle and the CC contact point are located at 1/2 of the length of their respective distal phalanges. The basic FingerPen posture (described here and shown in Fig. 1) has been chosen after some preliminary investigations aimed at identifying an arrangement of the fingers easy to be adopted and kept during time without effort. As a matter of fact, if, for instance, the middle finger was not located next to the index finger, but *on* the index finger - as some people tend to do - it would result in a posture causing physical fatigue, due to the weight of the middle finger that would load the index finger.

### The FreeFinger posture

In the FreeFinger (FF) posture, the position of the fingertip of the index is uniquely determined by the kinematic chain representing the index finger, which is a 4-DoF manipulator. Hence, the joint variables of the other fingers can be neglected, leading to $\mathbf {q}, \dot{\mathbf {q}}\in {\mathbb {R}}^4$. Thus, the fingertip velocity is described by

2 $$\begin{aligned} \dot{\mathbf {c}}_{he} = \mathbf {J}_i \dot{\mathbf {q}} \end{aligned}$$

where $\mathbf {J}_i \in {\mathbb {R}}^{3x4}$ is the Jacobian of the index finger, mapping the actual velocity of the manipulator’s joints in the velocity of the end-effector (index fingertip).

## Manipulability analysis

Manipulability analysis^40^ gives insights on the role played by the configuration of the manipulator in the map describing the relation between joints and end-effector velocities. More specifically, it allows to identify the directions along which the configuration of the manipulator amplifies the joint velocities and which are the motion directions along which the end-effector velocity is slowed down due to that manipulator configuration. The equation

3 $$\begin{aligned} \dot{\mathbf {q}}^T \dot{\mathbf {q}} = 1 \end{aligned}$$

describes a sphere in the joint velocity space. That set of joint velocities is mapped onto the end-effector operational space by accounting for the actual relation between joint velocities and end-effector velocities, that depends on the manipulator configuration. In “FingerPen manipulability” and “FreeFinger manipulability”, we will provide the manipulability analysis for the FingerPen and FreeFinger configurations, respectively. The analysis was conducted using the SynGrasp MATLAB Toolbox^41^.

### FingerPen manipulability

**Figure 3:**
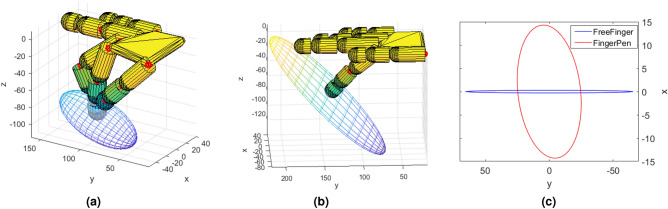
Manipulability analysis: Velocity ellipsoids describing the (**a**) FingerPen and (**b**) FreeFinger configurations. (**c**) Projection of the ellipsoids on the writing plane.

In the FingerPen configuration, Eq. (3) and Eq. (1) yield $\dot{\mathbf {c}}_{he}^T (\mathbf {J}_{eq}^{ \#})^T \mathbf {J}_{eq}^{ \#}\dot{\mathbf {c}}_{he} = 1$. However, it can be shown that $(\mathbf {J}_{eq}^{ \#})^T \mathbf {J}_{eq}^{\#}~=~ (\mathbf {J}_{eq}\mathbf {J}_{eq}^T)^{-1}$, leading to

4 $$\begin{aligned} \dot{\mathbf {c}}_{he}^T (\mathbf {J}_{eq}\mathbf {J}_{eq}^T)^{-1}\dot{\mathbf {c}}_{he} = 1 \end{aligned}$$

that describes how the set of points in Eq. (3) are mapped in an ellipsoid, whose semi-axes directions and dimensions are given by the eigenvectors and eigenvalues of the matrix $\mathbf {J}_{eq}\mathbf {J}_{eq}^T$, respectively. Eigenvectors characterize the orientation of the ellipsoid, while eigenvalues define the ellipsoid shape, encapsulating the information on the directions along which the end-effector is capable of higher and lower velocities. Results are visually shown in Fig. 3a.

### FreeFinger manipulability

In the FreeFinger configuration, Eq. (3) and Eq. (2) yield $\dot{\mathbf {c}}_{he}^T (\mathbf {J}_{i}^{\#})^T \mathbf {J}_{i}^{\#} \dot{\mathbf {c}}_{he} = 1$ that, thanks to the fact that $\mathbf {J}_{i}\mathbf {J}_{i}^T$ is a full-rank matrix, can be rewritten as

5 $$\begin{aligned} \dot{\mathbf {c}}_{he}^T (\mathbf {J}_{i}\mathbf {J}_{i}^T)^{-1}\dot{\mathbf {c}}_{he} = 1 \end{aligned}$$

Hence, ellipsoid semi-axes directions and lengths are given by the eigenvectors and eigenvalues of the matrix $\mathbf {J}_{i}\mathbf {J}_{i}^T$, respectively. Results are visually shown in Fig. 3b.

### Comparison on the Writing Plane

At first glance, Fig. 3 seems to suggest that the index fingertip is capable of faster motions along the transverse direction, both in the FP and in the FF configurations. However, what is worth to investigate is the actual motion capability of the fingertip on the writing plane. To this aim, the Cartesian, closed-form equation of the ellipsoids has been retrieved and intersected with the writing plane equation, leading to the equations of the ellipses resulting from the projection of the ellipsoids on the writing plane. For an easy visual comparison, such ellipses are drawn superimposed in Fig. 3c.

Despite an apparent similarity between the velocity ellipsoids of the FP and FF configurations, the actual motion capabilities of the index finger are remarkably different on the writing plane. As shown in Fig. 3c, indeed, the FingerPen configuration allows a larger velocity along the longitudinal direction, while in the FreeFinger configuration the finger can move faster in the transversal direction (the longer the ray vector, the higher is the allowed velocity along that direction). This argument allows a further consideration. During manipulation tasks, indeed, a trade-off between precision and velocity occurs, meaning that precise motions usually require low velocity, and high speed movements generate an imprecise control of the motion. This is due to the fact that in humans precise movements are the result of hand-eye coordination with continuous visual servoing providing proper feedback on the performed trajectory. The higher is the velocity, the less effective is the visual feedback to change the trajectory, due to a longer distance covered during a given time interval. To reflect this argument on Fig. 3c, we can say that the FingerPen allows a more precise motion along the transversal direction and a less precise motion in the longitudinal one. On the contrary, FreeFinger allows a precise motion along the longitudinal direction and a remarkably less precise motion along the transversal direction.

## Experiments

To have quantitative and qualitative evaluations on the actual use of the FingerPen for writing on touchscreens, we carried out an experimental campaign with 25 participants aged between 18 and 46. Two experiments were designed to retrieve quantitative data and, after the experiments, a questionnaire was submitted to the participants to distil insights on the user experience. As in “Manipulability analysis”, we carried out a comparative analysis between FingerPen and FreeFinger. Experiments investigated the relation between precision and workspace extension (see “Manipulability analysis”), and concerned the fact that the possibility of moving fast along certain directions usually leads to *i)* long traits and *ii)* imprecise movements. Hence, Experiment 1 focused on the extension of the occupied workspace when writing with a given hand posture (FP or FF), whereas Experiment 2 regarded the precision that can be achieved when using the FP and FF postures.

Participants were informed that they would perform experiments with two possible postures for writing on touchscreen devices, but no additional information on the idea behind the FingerPen was provided. The correct way to arrange the hand according to the FingerPen and FreeFinger configurations was shown to the participants by an expert, and they were let familiarize with the FingerPen configuration by performing free movements in the air for a couple of minutes. Informed consent was obtained from all subjects, no sensitive data were acquired and the other data were anonymized (GDPR 2016/679). All methods were carried out in accordance with relevant guidelines and regulations: In Italy, legislation that regulates the requirements for approval of experimental protocols by an Ethical Committee ((EU) 536/2014, (EU) 2017/745, Circular of the Italian Ministry of Health n.6 - 09-02-2002, Italian Ministerial Decree DM 03-18-1998) concerns clinical and pharmaceutical treatments only, hence this work (dealing with the common and safe task of writing on touchscreens) is exempted from approval by ethical committees. Participants were free to leave the experiments whenever they wanted, and the experimental protocols conformed with the principles inspiring the Declaration of Helsinki.

**Figure 4:**
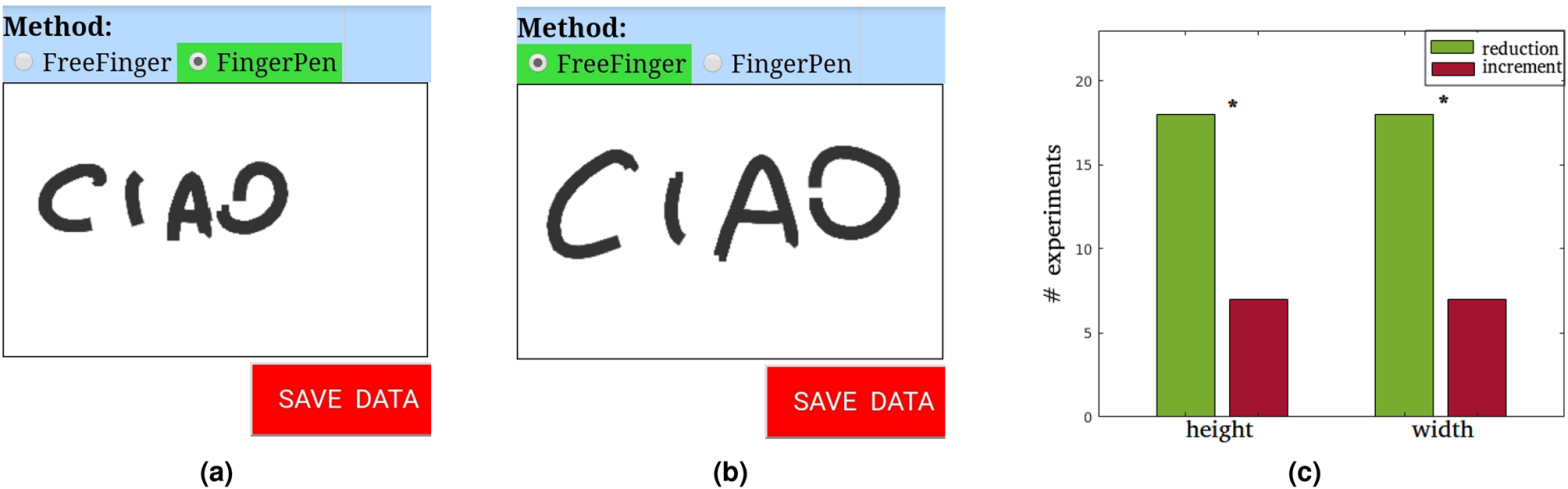
Experiment 1. On the left: example of the performed experiment aimed to investigate the space occupied when writing a simple word using (**a**) FingerPen and (**b**) FreeFinger. On the right (**c**), number of performed experiments leading to occupied workspace reduction/increment when using the FingerPen. The “*” symbol stands for “statistically significant difference with $p<0.05$”.

All the experiments required just a smartphone with touchscreen. Data generated by the user touch were acquired through a dedicated webapp implemented in HTML/PHP (see user interface in Fig. 4 and 6), and sent to a server where they were stored before data processing. Points from the performed finger trajectory were acquired at approximately 50 Hz.

In general, in handwriting tasks, size, speed and precision are highly subject-specific. Hence, we focused on the distribution of the individual differences to have insights on the effect of the FP and FF writing postures. Therefore, quantitative data gathered from all the participants underwent a statistical analysis in SPSS Statistics (Statistical Package for Social Science^42^, v.26) to check whether data acquired with FingerPen and FreeFinger differ in a statistically significant manner. If the individual differences were normally distributed (Shapiro-Wilk’s test, p > 0.05) and without extreme outliers (boxplot inspection), a paired samples T-test was conducted (statical significance for p < 0.05). When normality had been violated, a non-parametric test was conducted. To account also for effect size estimate, we considered the Cohen’s *d* coefficient, whose absolute value provides a measure of the signal-to-noise ratio (0.2: small, 0.5: medium, 0.8: large effect^43^).

### Experiment 1: investigation on the workspace

In this experiment, participants were asked to write the word “CIAO” (Italian word for “Hello”) in capital letters, within a rectangular box, two times: once using the FingerPen, and once using the FreeFinger. In Fig. 4a,b we show an example of this experiment: The area allowed for writing was defined by the solid, thin, black line.

Bearing in mind the considerations of the sociologists cited in “Introduction”, we deliberately asked people to write in Italian and not in English, because we believe that asking people to write in a language different from the native one (and not used in everyday life) may introduce some artefacts^44,45^.

Data acquired from the webapp were processed in MATLAB (v. 2019)^46^. Width and height of the text bounding box were retrieved for each participant and each hand configuration. A paired-samples T-test has been conducted to compare results for the FingerPen and FreeFinger configurations, revealing that data differ in a statistically significant manner ($$p<0.05$$) concerning both text width and height. Concerning height ($$p = 0.026$$, $$t=-2.38$$), the FingerPen allows a reduction of the maximum text height of about $$10.3 \pm 21.6$$ px, with medium effect size $$ d = - 0.5$$. Regarding text width ($$p=0.01$$, $$t = -2.77$$), the FingerPen allows a decrement of $$17.1 \pm 30.8$$ px, with effect size $$ d = - 0.6$$. As shown in Fig. 4c, the 72% of the experimenters experienced a reduction of the occupied workspace while using the FingerPen.

### Experiment 2: investigation on precision

**Figure 5:**
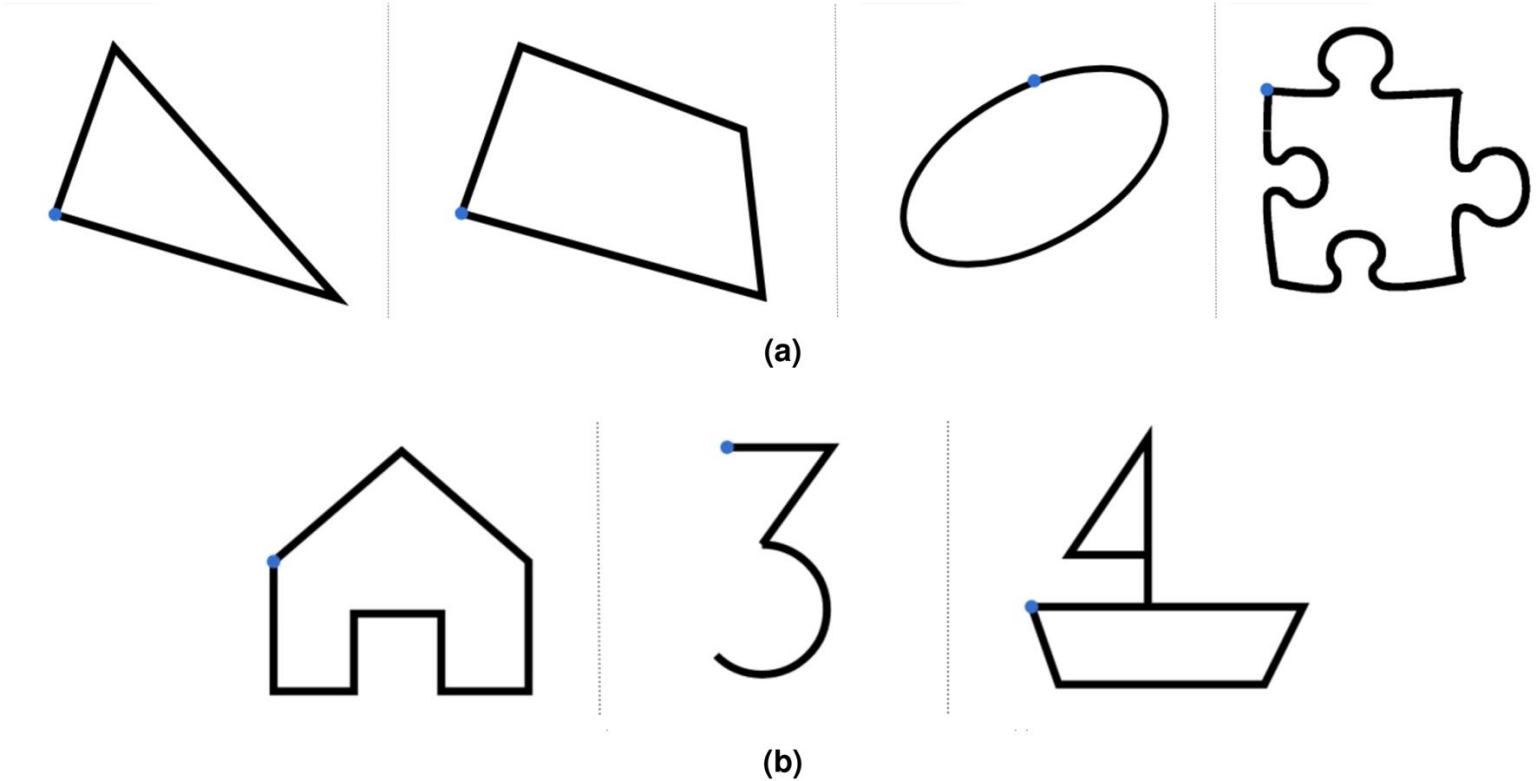
Experiment 2. (**a**) Figures for evaluation: triangle, quadrangle, ellipse, puzzle piece. (**b**) Figures for training: house, three, boat. The blue circle indicates the starting point.

**Figure 6:**
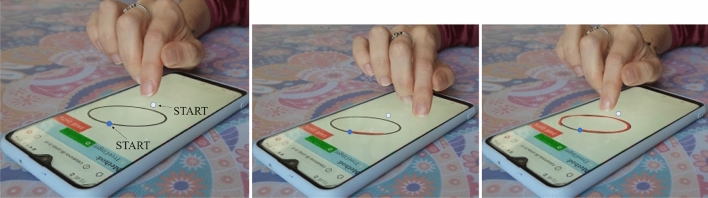
Experiment 2: intermediate steps. The blue circle indicates the starting point of the desired contour, whereas the white circle indicates the starting point of the tracing motion.

In this experiment, we investigated the precision that can be achieved by exploiting the FingerPen and FreeFinger postures. Participants were asked to trace the outline of different figures using the FP e FF. Four geometrical figures have been chosen on the basis of their shape (Fig. 5a): triangle (straight lines and sharp angles), quadrangle (straight lines, sharp and obtuse angles), ellipse (curved line with symmetry axes rotated with respect to the axes of the workspace), puzzle piece (straight and curved lines). To avoid local self-occlusions of the traced contour, we designed a displaced copying task (Fig. 6). Participants were provided with a canvas containing a geometrical figure in the upper part and a large blank area in the lower part. On the geometrical figure, there was a small circle coloured in blue: This point was matched with the corresponding, hollow, small circle located in the lower part of the canvas. Starting from this hollow circle, participants had to retrace the figure: Although the user touches the screen in points that are different from the location in which the geometrical figure is located, their trait appears in the region of the geometrical figure. In some sense, it is like to have an extension of the index finger, as it happens in some calligraphic tasks exploiting the oblique pen nib holder. In this way, users have a precise visual feedback on the precision related to their motion.

Since the FingerPen posture is somehow unusual for people, we wanted to investigate the effect of a small training on precision. To this aim, we designed three figures (house, number three, boat) that were thought to resemble the pre-writing exercises that people usually perform during childhood. We thought that engaging experimenters by stimulating memories capable of freeing the mind from the current performance would allow users to familiarize better with the FingerPen, leading also to a performance improvement. Figures used for training are shown in Fig. 5b.

Each experimenter carried out a session articulated in three steps: *i)* pre-training, *ii)* training; *iii)* post-training. During pre-training, the participant is asked to draw the triangle and the ellipse exploiting FingerPen and FreeFinger, alternately. Training was dedicated, instead, only to the FingerPen, and stopped when the participant felt at ease with the FingerPen posture; tracing at least three times per figure was recommended. During post-training, participants were asked to trace the quadrangle and the puzzle piece; such figures are slightly more complex than the ones used during pre-training. At the end of the session, three instances of each figure were drawn for each hand posture.

For each trial, the user’s trace and the time required to draw it were recorded. The former was saved as a sequence of *N* points $\mathbf {p}_i,\,i=1,\dots ,N$, the latter was computed as the interval between the first instant in which the user touched the screen and the instant in which the user lifted the finger from it.

For each gathered user’s contour, the mean error has been computed as the average of the distances between each user’s point ($\mathbf {p}_i$) and the corresponding nearest neighbour point ($\mathbf {n}\mathbf {n}_i$) belonging to the original contour ($e_i = ||\mathbf {p}_i - \mathbf {n}\mathbf {n}_i||$). In addition, we computed the maximum error ($e_{max} ~= ~\max ( \{e_i\}_{i=1,...,N} )$). These metrics were intended to be a first indicator of precision. For each contour, also the standard deviation of the error has been computed ($std ~= ~$std$\{[e_i]_{i=1,...,N}\}$) and considered as an indicator of regularity in the tracing task (i.e., the less is the standard deviation, the more regular is the task execution).

**Figure 7:**
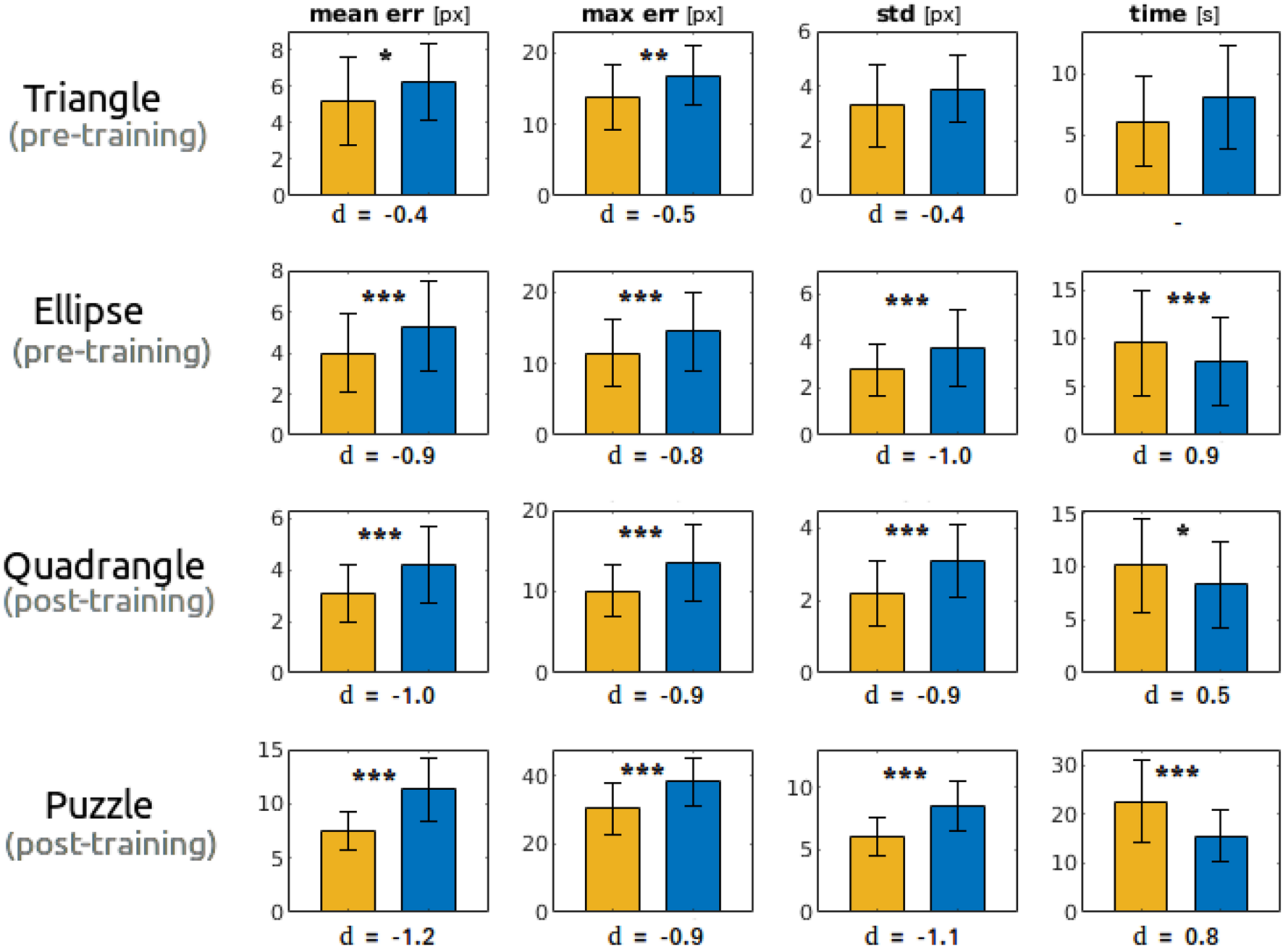
Experiment 2. Barplots of the descriptive statistics related to the metrics mean and max errors, std and time for each investigated figure. Yellow and blue colors are related to FingerPen and FreeFinger, respectively.

For each participant, all the data related to the same figure and acquired with the same hand posture were averaged to retrieve only one measure per person, figure and method. Such data underwent an analysis in SPSS for statistical significance of possible differences. A paired-samples T-test was conducted to investigate the distribution of the individual differences for all the figures and metrics except for the time in the triangle (a violation of normality occurred, and a Wilcoxon Sign test was performed). In Fig. 7, we show a representation of the results. Symbols ’*’, ’**’, ’***’ stand for “statistically significant difference with $p\le 0.05$, $p\le 0.01$, $p\le 0.001$”, respectively. *d* denotes the effect size according to the Cohen’s definition.

### Questionnaire

To have insights on the user experience, after the experiments the users filled out a questionnaire concerning aspects specifically related to the FingerPen configuration, and aspects related to the comparison between FingerPen and FreeFinger.

On the FingerPen configuration, the following questions were asked, and results are reported in parentheses:

- FP1: I felt difficult to keep the FP configuration: at the beginning (48%), during all the experiments (4%), no (48%).
- FP2: The difficulty I felt was especially: mental, physical (36%), both mental and physical, not relevant (64%).
- FP3: The training for FingerPen was: very useful (28%), quite useful (56%), a little useful (16%).
- FP4: Keeping the hand in the FingerPen configuration was: natural (56%), artificial (36%), neutral (8%).

**Figure 8:**
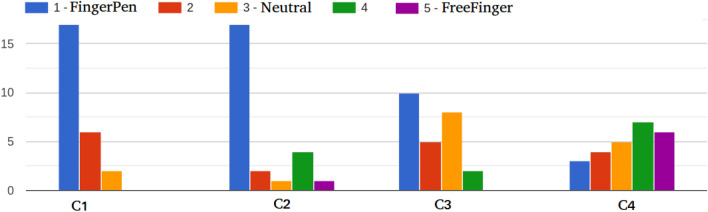
Answers to the questions on the user experience about the comparison of FingerPen and FreeFinger. Values 2 and 4 represent intermediate scores.

The questions on the comparison between FP and FF are reported in the following and results are shown in Fig. 8:

- C1: I believe that I was more precise while using...
- C2: I believe I had a greater control of the hand motions while using...
- C3: I found more pleasing the experience with...
- C4: I believe that the configuration inducing the most errors while tracing was...

## Discussion

### Comparison between FingerPen and FreeFinger postures

In “Manipulability analysis”, we retrieved a model-based characterization of the hand operational space while using FingerPen and FreeFinger. “Experiment 1: investigation on the workspace” showed that in the 72% of the trials the FingerPen allowed a reduction of the overall workspace occupied by the written text, and that the reduction was statistically significant both in height and width. Moreover, qualitatively speaking, the text usually appears more similar to what we are used to observe in adults (see also Fig. 4). The hypothesis we made on the basis of the manipulability analysis, i.e. that the FreeFinger would lead to a larger text, has been experimentally confirmed. However, the manipulability analysis provides information on the allowed velocity of the finger; but the actual length of traits depend on how much the manipulator configuration constrains the manipulator movements. Hence, in the FP case the occurring tripod grasp causes an overall reduction of the reachable planar workspace. Moreover, an additional explanation can be the fact that the extension of the space needed while writing is related to the effectiveness in the hand control while drawing letters. Hence, the better control provided by the FP contributes in reducing the overall size of the text.

Concerning precision (“Experiment 2: investigation on precision”, Fig. 7), a first consideration can be done on the difference between pre- and post- training. On average, the effect size in post-training is higher than in pre-training for all the metrics (as expected), suggesting that FP training leads to data distributions that differ more from the FF (see also the p-values). In the case of figures with effect size of similar magnitude (i.e., ellipse, quadrangle and puzzle, disregarding pre- and post-training), it can be noticed that the numerical difference between the values of the FP/FF metrics is larger as the figure is more complex (puzzle piece); however, the relative improvement is quite constant for each metric, independently on the figure complexity. In general, long straight traits lower the average error, and the main difficulty is near the angles or in the curved parts (see also results on *max err*). Data related to the *std* metric show that in general the FP allows a more regular trait, with less dispersion. As expected, the higher are precision and regularity, the longer is the time required to accomplish the task (see the *time* column).

Concerning the questionnaire (“Questionnaire”, Fig. 8), the vast majority of experimenters reported that if a difficulty was present during the experiments, it was related to the need of physically shaping the hand according to the FingerPen posture (FP2), and it was overcome while going on with experiments (FP1). This suggests that the difficulty was due to the need of getting used to another hand configuration, and is further supported by the fact that more than the 80% of the experimenters found the training step very or quite useful (FP3). Interestingly, the fact that the FingerPen configuration is somehow unusual for people (FP4) and requires some adaptation capabilities, does not impact negatively on the user experience: The vast majority of the experimenters had the perception of having greater control on the hand movements while using the FingerPen rather than the FreeFinger (C2). Moreover, they report that they felt more precise while exploiting FingerPen rather than FreeFinger (C1). Only two experimenters found the experience with the FreeFinger more pleasing (C3), and one half of the experimenters believe that the FreeFinger induces more in errors than the FingerPen, while the 20% of the experimenters found a tie on this aspect (C4). Hence, the analysis of the questionnaires strongly suggests that, from a user perspective, the advantages of adopting the FingerPen configuration for handwriting on touchscreen devices are remarkably more relevant than the disadvantages.

### Additional investigation: comparison between FingerPen and Pen postures

**Figure 9:**
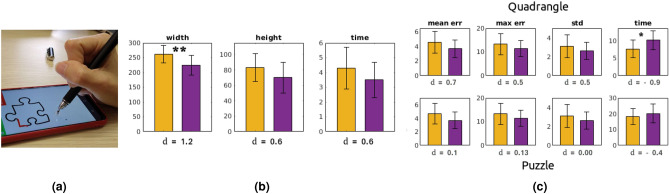
(**a**) Pen posture. (**b**), (**c**) Comparison between FingerPen (yellow) and Pen (purple): Investigation on workspace (**b**), and precision (**c**).

The focus of this work is on investigating hand postures for writing on touchscreens without using specific tools. Motivations are illustrated in “Introduction”. However, people are used to write with a stylus. Hence, for the sake of completeness, here we provide an experimental comparison of the performance achievable with FingerPen and Pen postures. The Pen posture corresponds to the hand configuration that is typically used to hold a stylus while writing^31^ (see, e.g., Fig. 9a). We asked 15 additional participants to perform the experiments on workspace and precision described in “Experiments” using a capacitive stylus. Figures for training (Fig. 5b) were used to let experimenters familiarize with the use of the pen in the proposed tasks before starting the experiments. Then, three instances of each figure shown in Fig. 5a were proposed alternately to each user. Data were collected and processed as previously described in “Experiments”, and a statistical analysis was carried out by performing independent T-tests.

Concerning the workspace occupancy (Experiment 1), the Pen allows a statistically significant reduction of the text width ($37.6\pm 11.4$, $p=0.003$, $t=3.3$), see Fig. 9b. Also the text height and the completion time are reduced, although without statistical significance. Concerning the experiments on precision (Experiment 2), in Fig. 9c we show the results on the quadrangle and puzzle figures. In general, the task completion time is slightly higher for the Pen, whereas the metrics related to the errors are slightly lower for the Pen than for the FingerPen. The difference is even more negligible for the most complex figure (puzzle). Results on the comparison performed on triangle and ellipse show the same trend as the quadrangle.

In the following lines, we provide a possible interpretation of the obtained results, taking into account that the performance with the stylus is affected by the users’ familiarity with the tool. Results on the occupancy of the workspace are consistent with what was expected: the Pen allows tighter and faster writing, although only the width reduction is statistically significant, and with remarkable effect size. In the experiments on precision, we expected a shorter completion time for the Pen, differently from what was obtained in the experiments. However, this can be due two main factors. First, there are two displacements to be considered: $D_{p,t}$, i.e., the distance between the pen tip and the point where the trait actually appears (see Fig. 6, displaced copy task), and $D_{p,f}$, i.e., the distance between the pen tip and the points where the pen is grasped by the fingers. These two distances make the task with the stylus more demanding than using the FingerPen, because not only $D_{p,t}$, but also $D_{p,f}$ impact the user’s tracking of the figures. As a consequence, the visual servoing that the user performs to track a certain profile becomes more challenging, requiring a careful control of the forces and torques applied to the writing tool. Second, not everyone is equally familiar with the adoption of a stylus to interact with touchscreens (and smartphones in particular), whereas all of us are used to interact with screens through our fingers. Hence, it can be reasonably argued that the above mentioned factors partially reduce user’s familiarity and ability with styluses, resulting in a task performance with a slightly higher precision at the cost of a higher task completion time.

## Conclusions

In this paper, we proposed the FingerPen, a novel hand posture that can be adopted for writing on touchscreens without the use of specific input devices (e.g., a stylus). By exploiting modeling tools from biomechanics and the theory of grasping, a characterization of the workspace of this posture as been provided. A comparison with the hand posture that people typically adopt for interacting with touchscreens, i.e., the free index finger (FreeFinger), showed that FingerPen allows a greater control of the hand and more precise motions. The conducted experimental campaign with 25 participants confirmed the model-based results, and a questionnaire on the user experience reported a strongly positive opinion on the FingerPen adoption.

Future work will focus on the analysis of the FingerPen performance when adopted by experimenters of different age groups, with particular attention to children and elders. We will investigate more in detail the impact on the graphomotor execution and the features of the traits (such as smoothness, number of strokes, writing slant and curvature). A larger spectrum of experiments will be designed, considering also the impact that the use of the FingerPen can have on writing recognition systems^47,48^.
